# Carotid Web as a Source of Thromboembolism in a Young African American Female

**DOI:** 10.31486/toj.23.0082

**Published:** 2024

**Authors:** Eric Assid, Chad Hall, Mawadah Samad, Richard Zweifler

**Affiliations:** ^1^ Department of Sports Medicine, Ochsner Clinic Foundation, New Orleans, LA; ^2^ School of Medicine, Tulane University, New Orleans, LA; ^3^ The University of Queensland Medical School, Ochsner Clinical School, New Orleans, LA; ^4^ Department of Neurology, Ochsner Clinic Foundation, New Orleans, LA

**Keywords:** *Carotid artery diseases*, *carotid sinus*, *stroke*, *thromboembolism*

## Abstract

**Background:** Carotid webs are nonatherosclerotic fibrous bands that may alter hemodynamic flow and increase the risk of platelet aggregation, leading to thromboembolism in young, otherwise healthy individuals. Although rare, carotid webs are important causes of thromboembolic strokes and are often overlooked in the routine workup for a stroke. Treating physicians and radiologists must recognize and properly manage patients who present with carotid webs to prevent recurrent thromboembolism.

**Case Report:** A healthy 30-year-old female presented with slurred speech and unilateral left upper and lower extremity numbness. Imaging modalities showed an acute infarction of the right middle cerebral artery and bilateral carotid webs. The patient was managed operatively with a right carotid endarterectomy and discharged on day 3 of admission on a regimen of ticagrelor, amlodipine, and aspirin. The patient was asymptomatic at 1-year follow-up.

**Conclusion:** Our case highlights the clinical relevance of considering carotid web as a potential etiology for ischemic stroke in young, otherwise healthy patients and emphasizes the importance of timely diagnosis and appropriate management to prevent recurrent cerebrovascular events.

## INTRODUCTION

Carotid webs are nonatherosclerotic fibrous bands that appear on imaging modalities as thin intraluminal filling defects along the posterior wall of the carotid bulb cephalad to the carotid bifurcation.^1^ Considered a potential variant of fibromuscular dysplasia, carotid webs are characterized histopathologically by fibroelastic thickening of the arterial tunica intima.^1^ These fibrous bands may alter hemodynamic flow and increase the risk of platelet aggregation, leading to thromboembolism in young, otherwise healthy individuals.^2^ Carotid webs are important to identify and treat as they are a major preventable risk factor for recurrent thromboembolism. Because of the close association of carotid webs with fibromuscular dysplasia, patients may experience headaches; tinnitus; and pain in the neck, flank, and abdomen.^3,4^ When carotid webs are themselves symptomatic, focal symptomatology of strokes may occur.^4^ The prevalence of carotid webs is still being studied in large populations, but a retrospective study published in 2015 estimated the prevalence to be 1.2% of patients admitted to the hospital with suspected stroke.^5^ This study only accounted for hospitalized patients, however, and was not a large population-based study.

Computed tomography angiography (CTA), magnetic resonance angiography, and conventional contrast angiography are all imaging modalities of choice for diagnosis and discussion of management. Carotid webs should be suspected in young, otherwise healthy patients who present with stroke and do not have conventional risk factors for recurrent thromboembolism.

## CASE REPORT

A 30-year-old, nonsmoking, gravida 1, para 1, African American female, 10 months postpartum, with a history of obesity (body mass index 43.3 kg/m^2^) and a family history of stroke presented to the emergency department (ED) for left upper and lower extremity numbness and slurred speech. The patient reported a last known normal approximately 1 hour prior to arrival at the ED. Symptom onset occurred while the patient was in the sauna at the gym. She first noticed left upper and lower extremity numbness that progressed to include slurred speech while she attempted to call a family member. By the time the patient arrived at the ED, her symptoms had improved. On physical examination, she was afebrile with blood pressure 122/58 mm Hg, pulse 84 beats/min, and blood oxygenation 98%. Speech problems and lower extremity numbness were not present, but the left upper extremity hypoesthesia remained. The patient was alert and oriented to person, place, and time. She had a Glasgow Coma Scale score of 15, no focal motor deficits were noted, and cranial nerves II-XII were intact. The patient's head was normocephalic and atraumatic. Her cardiovascular examination was normal, pulmonary examination showed normal pulmonary effort, and musculoskeletal examination showed no deformities.

**Figure 1.**
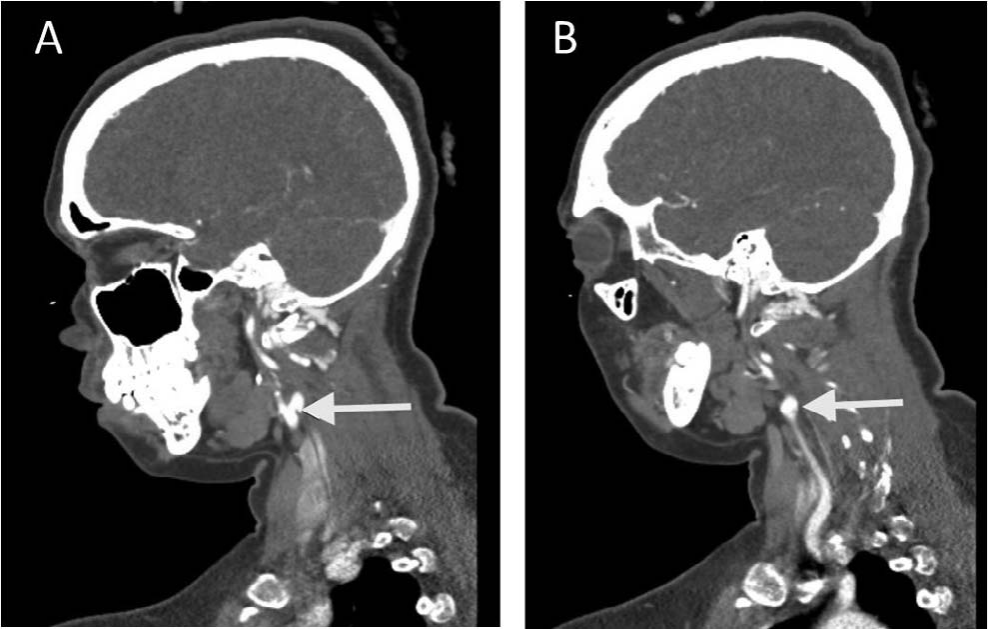
Computed tomography angiography images showing (A) the left carotid bifurcation and (B) the right carotid bifurcation.

**Figure 2.**
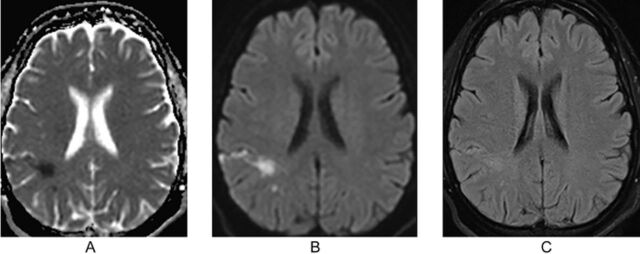
(A) Apparent diffusion coefficient showing hypointense diffusion restriction in the right posterior frontal lobe. (B) Diffusion-weighted image showing hyperintense diffusion restriction in the right posterior frontal lobe. (C) T2 fluid attenuation inversion recovery without contrast showing an infarct in the posterior frontal lobe within the right middle cerebral artery territory.

CTA multiphase showed no acute abnormalities, no high-grade stenosis, and no major vessel occlusion. Incidental bilateral carotid bifurcation webs were discovered (Figure 1). Magnetic resonance imaging of the brain showed a band-like acute infarct involving the posterior aspect of the right frontal lobe extending into the periventricular white matter (Figure 2). Transthoracic echocardiogram showed an ejection fraction of 60%, pulmonary artery pressure of 25 mm Hg, normal ventricle size and function, and a small mobile density on the ventricular aspect of the tricuspid valve. The differential diagnosis for this finding includes papillary fibroelastoma or vegetation. No intracardiac shunt was identified with the saline bubble test.

The patient's laboratory values were hemoglobin 10.9 g/dL (reference range, 12.0-16.0 g/dL), hematocrit 36.4% (reference range, 37.0%-48.5%), potassium 3.0 mmol/L (reference range, 3.5-5.1 mmol/L), chloride 111 mmol/L (reference range, 95-110 mmol/L), CO_2_ 19 mmol/L (reference range, 23-29 mmol/L), calcium 8.4 mg/dL (reference range, 8.7-10.5 mg/dL), and high-density lipoprotein 39 mg/dL (reference range, 40-75 mg/dL).

Initial treatment in the ED included aspirin 81 mg and clopidogrel 75 mg. The patient also received a subcutaneous heparin injection of 5,000 units every 8 hours throughout her hospitalization.

The patient had complete resolution of symptoms, with a National Institutes of Health Stroke Scale score of zero on the second day of admission. After other etiologies were ruled out, the stroke was determined to most likely be secondary to embolism from the right carotid web. On day 2 of admission, the patient was managed operatively with a right carotid endarterectomy to prevent future thromboembolic strokes. The patient was discharged on day 3 of admission with ticagrelor 90 mg 2 times daily, amlodipine 5 mg once daily, and aspirin 81 mg once daily. Of note, she was discharged on ticagrelor because of a normal platelet function assay on clopidogrel 75 mg/day.

The patient was instructed to follow up with vascular surgery for a bilateral carotid ultrasound in 4 weeks and to follow up with internal medicine and vascular neurology. CTA at 1-year follow-up demonstrated evidence of right-sided endarterectomy without evidence of stenosing and a small stable left carotid web with a patent left common carotid artery. The patient remains asymptomatic and continues to take ticagrelor.

## DISCUSSION

Our case was unique as the patient had no previous diagnosis of carotid webs, fibromuscular dysplasia, or other angiopathies. In addition, she had no risk factors (she was young and did not smoke), and standard genetic testing for COL5A1, a commonly mutated gene in fibromuscular dysplasia, was negative. The patient received interventional care to reduce the right carotid web and remains on ticagrelor with no further evidence of ischemic attacks.

Turbulence and stagnation of blood downstream of the carotid web are thought to increase the risk for thrombus formation and subsequent embolism.^2^ Recognizing risk factors, such as recent pregnancy and obesity, associated with thrombus formation is important.

Findings of a 2018 systematic review showed that 67% of patients with symptomatic carotid webs were female and 70% were of African race.^4^ Joux et al found that carotid webs increase the risk of ischemic strokes in Afro-Caribbean populations,^6^ while Haussen et al reported that 61% of patients with carotid webs in their study were females and 75% were Black.^7^ Optimal management of carotid webs remains uncertain, as the prevalence is low and no prospective, randomized studies have been conducted.^5^ Rates of recurrence vary depending on the method of treatment. In their nonrandomized study, Olindo et al reported a 27.3% 5-year ipsilateral stroke or transient ischemic attack recurrence rate in patients treated solely with antithrombotic therapy vs no recurrent events in patients treated with either stenting or endarterectomy.^8^

## CONCLUSION

This case emphasizes the clinical significance of carotid webs as a potential cause of ischemic stroke in young, otherwise healthy individuals and demonstrates the importance of early recognition with appropriate management to prevent recurrent thromboembolism. This case is a reminder for physicians and radiologists to consider carotid webs as a possible etiology in the absence of conventional risk factors. Furthermore, this case demonstrates the need for further research to determine optimal management for carotid webs. Prospective randomized trials are warranted to establish evidence-based recommendations for the management of symptomatic carotid webs.

## Notes

*This article meets the Accreditation Council for Graduate Medical Education and the American Board of Medical Specialties Maintenance of Certification competencies for Patient Care and Medical Knowledge.*
